# Potential of a fucoidan-rich *Ascophyllum nodosum* extract to reduce *Salmonella* shedding and improve gastrointestinal health in weaned pigs naturally infected with *Salmonella*

**DOI:** 10.1186/s40104-022-00685-4

**Published:** 2022-04-04

**Authors:** Brigkita Venardou, John V. O’Doherty, Shane Maher, Marion T. Ryan, Vivian Gath, Rajeev Ravindran, Claire Kiely, Gaurav Rajauria, Marco Garcia-Vaquero, Torres Sweeney

**Affiliations:** 1 School of Veterinary Medicine, University College Dublin, Belfield, Dublin 4 Ireland; 2 School of Agriculture and Food Science, University College Dublin, Belfield, Dublin 4 Ireland

**Keywords:** *Ascophyllum nodosum*, Gastrointestinal microbiota, Inflammation, Pig, *Salmonella*, Seaweed extract, Zinc oxide

## Abstract

**Background:**

Dietary supplementation with a fucoidan-rich *Ascophyllum nodosum* extract (ANE), possessing an in vitro anti-*Salmonella* Typhimurium activity could be a promising on-farm strategy to control *Salmonella* infection in pigs. The objectives of this study were to: 1) evaluate the anti-*S.* Typhimurium activity of ANE (containing 46.6% fucoidan, 18.6% laminarin, 10.7% mannitol, 4.6% alginate) in vitro, and; 2) compare the effects of dietary supplementation with ANE and Zinc oxide (ZnO) on growth performance, *Salmonella* shedding and selected gut parameters in naturally infected pigs. This was established post-weaning (newly weaned pig experiment) and following regrouping of post-weaned pigs and experimental re-infection with *S. *Typhimurium (challenge experiment).

**Results:**

In the in vitro assay, increasing ANE concentrations led to a linear reduction in *S. *Typhimurium counts (*P* <  0.05). In the newly weaned pig experiment (12 replicates/treatment), high ANE supplementation increased gain to feed ratio, similar to ZnO supplementation, and reduced faecal *Salmonella* counts on d 21 compared to the low ANE and control groups (*P* <  0.05). The challenge experiment included thirty-six pigs from the previous experiment that remained on their original dietary treatments (control and high ANE groups with the latter being renamed to ANE group) apart from the ZnO group which transitioned onto a control diet on d 21 (ZnO-residual group). These dietary treatments had no effect on performance, faecal scores, *Salmonella* shedding or colonic and caecal *Salmonella* counts (*P* > 0.05). ANE supplementation decreased the Enterobacteriaceae counts compared to the control. Enterobacteriaceae counts were also reduced in the ZnO-residual group compared to the control (*P* <  0.05). ANE supplementation decreased the expression of interleukin 22 and transforming growth factor beta 1 in the ileum compared to the control (*P* <  0.05).

**Conclusions:**

ANE supplementation was associated with some beneficial changes in the composition of the colonic microbiota, *Salmonella* shedding, and the expression of inflammatory genes associated with persistent *Salmonella* infection.

**Supplementary Information:**

The online version contains supplementary material available at 10.1186/s40104-022-00685-4.

## Background

Weaning is a critical period in pig production as the associated nutritional, emotional, social and environmental stressors reduce feed intake and increase gastrointestinal dysfunction and dysbiosis [1–4]. These changes result in reduced growth performance and increased susceptibility to pathogens including *Salmonella enterica* subsp. *enterica* serotypes. Previous studies have demonstrated that weaned pigs disseminate and maintain *Salmonella* infection at farm level [5–8]. Movement to grower and finisher houses, handling and re-grouping are additional stress factors that could increase *Salmonella* shedding and susceptibility to infection resulting in further pig-to-pig and contaminated environment-to-pig transmission on farms [6, 9]. Dietary supplementation with ZnO at pharmacological doses (2000–3000 mg/kg feed) during the immediate post-weaning period is a common practice to alleviate the negative impact of weaning on pig performance [10] and gastrointestinal functionality and health [11–13]. However, high ZnO inclusion levels in the pig diet have been associated with antimicrobial resistance (AMR) within the bacterial populations of the gastrointestinal microbiota [14–16].

The use of feed additives has been considered as a potential on-farm strategy to control *Salmonella* infection in pigs [17]. Brown seaweeds or macroalgae are a promising source of dietary non-digestible polysaccharides such as fucoidan, a structural component of the macroalgal cell wall, that has exhibited antibacterial [18, 19], prebiotic [20, 21] and immunomodulatory [22, 23] potential. Fucoidans are a heterogenous family of fucose-containing sulphated polysaccharides with a backbone structure consisting of α-(1 → 3)-linked or alternating α-(1 → 3) and α-(1 → 4)-linked L-fucopyranosyls [24]. Preliminary in vitro studies identified various fucoidan-rich seaweed extracts which inhibit the growth of the pathogenic *S. *Typhimurium or stimulate the growth of the commensal *Lactobacillus* spp. and *Bifidobacterium* spp. strains [25–28]. Furthermore, in an experimental infection with *S. *Typhimurium, dietary supplementation of pigs with a fucoidan-rich seaweed extract was associated with improved performance, reduced *Salmonella* shedding and colonisation and reduced intestinal inflammation [29]. However, the effects of fucoidan-rich seaweed extracts on pig performance and gastrointestinal health under a natural *Salmonella* infection have not been previously investigated.

Brown seaweed *Ascophyllum nodosum* contains 6.5–11.6% fucoidan and, thus, is commonly used as a source of this polysaccharide [30, 31]. The concentration, structure and bioactivity of fucoidan contained in the seaweed extracts is highly dependent on the extraction conditions [32, 33]. Hence, the first objective of this study was to evaluate the anti-*S.* Typhimurium activity of a fucoidan-rich *A. nodosum* extract (ANE) obtained using a hydrothermal-assisted extraction method in a pure culture growth assay to determine the two inclusion levels of ANE for the subsequent experiments. The second objective of this study was to evaluate the effects of dietary supplementation with two inclusion levels of ANE and the pharmacological level of ZnO on growth performance and *Salmonella* shedding in naturally infected weaned pigs during the first 21 d post-weaning (newly weaned pig experiment). The third objective of this study was to determine the effects of the best performing ANE inclusion level and the residual effects of ZnO on growth performance, *Salmonella* shedding, colonic and caecal *Salmonella* counts, the composition of the colonic microbiota, and selected inflammatory markers in the ileum and colon following an experimental re-infection with *S.* Typhimurium after pig transfer to the grower houses and regrouping (challenge experiment).

## Materials and methods

### *Ascophyllum nodosum* extract (ANE) preparation and chemical composition analyses

*A. nodosum* was harvested in February 2019 (Quality Sea Veg Ltd., Burtonport, Co. Donegal, Ireland). Whole seaweed biomass was oven-dried at 50 °C for 9 d and milled to a 1 mm particle size (Christy and Norris Hammer Mill, Chelmsford, UK) and stored at room temperature. The ANE extract was obtained using a hydrothermal-assisted extraction method using the optimal conditions for best fucoidan yield (120 °C, 62.1 min, 30 mL 0.1 mol/L HCl/g seaweed) as described previously [34].

The ANE composition as % w/w dry matter was as follows: 46.6% fucoidan, 18.6% laminarin, 10.7% mannitol, 4.6% alginate, 4.5% protein and 0.75% ash. The ANE was stored at − 20 °C. The concentration of fucoidan was estimated according to the method described by Usov et al. [35], with modifications as described by Garcia-Vaquero et al. [34]. The concentration of laminarin and mannitol was determined using standard kits (Megazyme Ltd., Bray, Co. Wicklow, Ireland) according to the manufacturer’s instructions. The concentration of alginate was estimated according to the method described by Truus et al. [36]. The ash content was determined after ignition of a weighed sample in a muffle furnace (Nabertherm GmbH, Lilienthal, Germany) at 550 °C for 6 h according to the AOAC.942.05 [37]. The nitrogen content was determined using the LECO FP 528 instrument (Leco Instruments UK Ltd., Cheshire, UK) according to the AOAC.990.03 [37]. The conversion factor 4.17 was used to calculate protein content, as described for brown macroalgae [38].

### In vitro screening of ANE antibacterial activity

The revival and culture of the *S.* Typhimurium phage type (PT) 12 and *Bifidobacterium thermophilum* (DSMZ 20210) and the subsequent pure culture growth assays were carried out as described by Venardou et al. [39]. Briefly, *S.* Typhimurium and *B. thermophilum* were revived from cryoprotective beads (TS/71-MX, Protect Multi-purpose, Technical Service Consultants Ltd., Lancashire, UK) and sub-cultured following standard procedures to obtain 24 h cultures. The pure culture growth assays were carried out in 96-well microtiter plates (CELLSTAR, Greiner Bio-One, Kremsmünster, Austria). ANE was diluted appropriately in 10% de Man, Rogosa and Sharpe broth (MRS, Oxoid Ltd., Hampshire, UK) and 10% Tryptone soya broth (TSB, Oxoid Ltd., Hampshire, UK) to obtain a final concentration of 5, 4, 3, 2 and 1 mg/mL prior to the assay. *S.* Typhimurium and *B. thermophilum* were diluted in 10% TSB and MRS, respectively, to obtain an inoculum of 10^6^–10^7^ CFU (Colony-forming unit)/mL with initial bacterial enumeration performed each time. Equal quantities of each ANE concentration and inoculum were transferred to duplicate wells and control wells containing no ANE were also included. To evaluate the sterility, blank wells containing equal quantities of 10% medium and each ANE concentration were included. Plates were agitated gently for thorough mixing and incubated at 37 °C for 18 h aerobically for *S.* Typhimurium or anaerobically for *B. thermophilum*. After incubation, a 10-fold serial dilution (10^−1^–10^−8^) followed by spread plating onto Tryptone soya agar (Oxoid Ltd., Hampshire, UK) for *S.* Typhimurium and de Man, Rogosa and Sharpe agar (Oxoid Ltd., Hampshire, UK) for *B. thermophilum* were used to determine both the bacterial viability and counts at the increasing ANE concentrations. Plates were incubated aerobically at 37 °C for 24 h for *S.* Typhimurium and anaerobically at 37 °C for 48 h for *B. thermophilum*. Anaerobic conditions were established within sealed containers using AnaeroGen 2.5 and 3.5 L sachets (Thermo Fisher Scientific, Waltham, MA, USA). The dilution resulting in 5–50 colonies was selected for the calculation of CFU/mL using the formula CFU/mL = Average colony number × 50 × dilution factor. The bacterial counts were logarithmically transformed (logCFU/mL) for the subsequent statistical analysis. Zero counts at the neat dilution (10^0^) were assigned the arbitrary value of 1.30 logCFU/mL which was considered the minimum detection limit using spread plating [40]. All experiments were carried out with technical replicates on three independent occasions (3 biological replicates).

### Newly weaned pig experiment (d 0–21)

#### Experimental design and diets

The experiment had a randomised complete block design and consisted of the following dietary treatments: (T1) basal diet (control); (T2) basal diet + 3.1 g ZnO (pharmacological dose)/kg feed (ZnO); (T3) basal diet + 2 g ANE/kg feed (low ANE) and (T4) basal diet + 4 g ANE/kg feed (high ANE). The ANE inclusion levels were selected based on the in vitro anti-*S. *Typhimurium activity of the 2 and 4 mg/mL ANE. In particular, the concentration of 2 mg/mL was the lowest ANE concentration with some anti-*S. *Typhimurium activity, whereas the concentration of 4 mg/mL ANE, along with 5 mg/mL, had the strongest effect. Ninety-six healthy pigs [progeny of meat-line boars × (Large White × Landrace sows)] with average weight 8.6 (standard deviation (SD) 1.12) kg were sourced from a commercial pig farm at weaning (28 days of age) and were penned in groups of two. At the time of weaning, the *Salmonella* seroprevalence for the herd in the farm of origin was estimated at 46.7% (weighted average of previous three months data). The pigs were blocked based on weaning weight, litter of origin and sex and within each block assigned to one of the four treatments (12 replicates/treatment). The basal diet contained 10.6 MJ/kg net energy and 14.0 g/kg standard ileal digestible lysine. All amino acid requirements were met relative to lysine [41]. The ingredient composition and the analysed and calculated chemical composition of the diet are presented in Table 1. All treatment diets were milled on site and fed in meal form for 21 d. The ZnO (Cargill, Naas, Co. Kildare, Ireland) was included at 3100 mg/kg feed and contained 80% Zn, resulting in an inclusion level of 2500 mg Zn per kg feed.

**Table 1:** Ingredient composition and chemical analysis of the basal diet^a^

**Ingredient, g/kg**	
Wheat	355.4
Full fat soya bean	170.0
Soya bean meal	105.0
Flaked wheat	130.0
Flaked maize	70.0
Soya oil	30.0
Soya concentrate	65.0
Whey powder (90%)	50.0
Vitamins and minerals^b^	2.5
Sodium bicarbonate	2.0
Monocalcium phosphate	4.0
Calcium carbonate (Limestone)	6.0
Salt	2.0
Lysine HCl	4.0
DL-methionine	2.0
L-threonine	1.8
Tryptophan	0.3
**Analysed and calculated chemical composition**	
Dry matter	899.0
Crude protein (N × 6.25)	208.0
Gross energy, MJ/kg	16.9
Crude fat	80.0
Crude fibre	28.0
Ash	46.0
Neutral detergent fibre	99.0
Lysine, %^c^	1.43
Methionine, %^c^	0.50
Methionine and cysteine, %^c^	0.84
Threonine, %^c^	0.93
Tryptophan, %^c^	0.30
Valine, % ^c^	0.98
Leucine, % ^c^	1.45
Isoleucine, % ^c^	0.87

^a^Dietary treatments: (T1) basal diet (control); (T2) basal diet + 3.1 g ZnO/kg feed (ZnO); (T3) basal diet + 2 g ANE/kg feed (low ANE); (T4) basal diet + 4 g ANE/kg feed (high ANE)

^b^Provided (mg/kg complete diet): Cu from copper sulphate, 25; Fe from ferrous sulphate monohydrate, 140; Mn from manganese oxide, 47; Zn from zinc oxide, 120; I from potassium iodate, 0.6; Se from sodium selenite, 0.3; retinol, 1.8; cholecalciferol, 0.025; tocopherol, 67; menaquinone, 4; cyanocobalamin, 0.01; riboflavin, 2; nicotinic acid, 12; pantothenic acid, 10; choline chloride, 250; thiamine, 2; pyridoxine, 0.015

^c^Calculated for tabulated nutritional composition [42]

#### Housing and animal management

The pigs were housed in fully slatted pens (1.7 m × 1.2 m) and weighed at the beginning of the experiment (d 0) and on d 7, 14 and 21. The ambient environmental temperature within the house was thermostatically controlled at 30 °C for the first 7 d and reduced by 2 °C per week for the remainder of the experiment. The humidity was maintained at 65%. Feed and water were available ad libitum from four-space feeders and nipple drinkers; precaution was taken to avoid feed wastage. Faecal scores (FS) were recorded twice daily in the individual pens by the same operator on a scale ranging from 1 to 5. The scoring system was as follows: 1 = hard, firm faeces; 2 = slightly soft faeces; 3 = soft, partially formed faeces; 4 = loose, semi-liquid faeces; and 5 = watery, mucous like faeces [43].

#### Sample collection for *Salmonella* presence and quantification

Faecal samples were collected after natural defaecation into sterile containers (Sarstedt AG & Co. KG, Nümbrecht, Germany) on arrival on d 0 from 19 pigs to determine the *Salmonella* status of the herd. Rectal faecal samples were collected into sterile containers from the same pig in each pen (*n* = 48 pigs) on d 14 and 21. Samples were obtained by natural defaecation with rectal stimulation employed only if necessary and solely on d 21. All faecal samples were immediately stored at − 20 °C.

### Challenge experiment (d 25–34)

#### Experimental design and diets

On d 21, ZnO supplementation ceased and one pig from each pen from (T1), (T2) and (T4) of the newly weaned pig experiment (*n* = 36, 12 replicates/treatment) proceeded to the challenge experiment. The pigs from (T1) and (T4) were kept on their original diets as described in the newly weaned pig experiment with (T4) being renamed as ANE, whereas (T2) was renamed as ZnO-residual, whereby the animals were fed a basal diet upon ZnO removal. Between d 21 and 25, all pigs were on their respective diet, however, performance data was collected after the initiation of the challenge experiment on d 25. The challenge experiment had a randomised complete block design. The thirty-six pigs with an average weight of 18.3 (2.44 SD) kg on d 25 were blocked on weight basis and penned in pairs.

#### Housing and animal management

The pigs were weighed at the beginning (d 25) and end (d 34) of the experiment. The housing and animal management were as described in the newly weaned pig experiment apart from the ambient environmental temperature that was kept at 25 °C during the nine-day experimental period in each house and the FS that was recorded once daily in the individual pens.

#### *S.* Typhimurium experimental infection

On d 25, each animal was manually restrained and orally challenged with 5 mL of a *S.* Typhimurium culture (infectious dose ≈ 4 × 10^7^ CFU) using a syringe (no needle attached).

#### Sample collection

Rectal faecal samples were collected in sterile containers from all pigs on d 25 (prior to *S.* Typhimurium infection), 27 and 34 for *Salmonella* quantification and immediately stored at − 20 °C. Samples were obtained following natural defaecation or if necessary with rectal stimulation. On d 34, all 36 pigs were euthanised by pentobarbitone sodium (Euthatal Solution, 200 mg/mL; Merial Animal Health, Essex, UK) overdose (1 mL/kg body weight injected into the cranial vena cava). Euthanasia was completed by a competent person in a separate room away from sight and sound of the other pigs. The entire intestinal tract was immediately removed. Colonic and caecal digesta were collected in sterile containers, snap frozen on dry ice and stored at − 20 °C for bacterial quantification using quantitative real time polymerase chain reaction (QPCR). Additionally, 1 cm^2^ sections from the ileum (15 cm from ileocaecal junction) and colon were removed, emptied by dissection along the mesentery and rinsed using sterile phosphate buffered saline (Sigma-Aldrich, St. Louis, MO, USA). The tissue sections were stripped of the overlying smooth muscle before overnight storage in 5 mL RNAlater® solution (Sigma-Aldrich, St. Louis, MO, USA) at 4 °C. The RNAlater® was removed before storing the samples at − 80 °C. These ileal and colonic tissue samples were used for gene expression analysis.

### Feed analyses

The feed was milled through a 1-mm screen (Christy and Norris Hammer Mill, Chelmsford, England). The dry matter content was determined after drying overnight at 104 °C. Ash content was determined after ignition of a weighted sample in a muffle furnace at 550 °C for 6 h according to the AOAC.942.05 [37]. The gross energy content was determined using an adiabatic bomb calorimeter (Parr Instruments, Moline, IL, USA). Crude protein content was determined by measuring the nitrogen content of the feed samples using the LECO FP 528 instrument and the conversion factor of 6.25 according to the AOAC.990.03 [37]. The neutral detergent fibre content was determined according to the method of Van Soest et al. [44] and the crude fibre content according to the AOAC method [37]. The crude fat content of the diets was determined using light petroleum ether and Soxtec instrumentation (Tecator, Sweden) according to the AOAC.920.39 [37].

### *Salmonella* isolation and serotyping

Faecal samples from d 0 were screened for the presence or absence of *Salmonella* in accordance with the protocol of the International Organisation for Standardization (ISO) 6579–1:2017. *Salmonella* serotyping, which involved agglutination tests with hyperimmune antisera specific for a range of somatic (O) and flagellar (H) antigens and comparison with the White-Kauffmann-Le Minor scheme [45], was also performed on *Salmonella* positive samples in accordance with ISO protocol 6579–3:2014. Isolates with a phenotypic partial serotyping were further analysed using a multiplex QPCR for differentiating *S.* Typhimurium and its monophasic variant *S.* 4,[5],12:i:- as described previously [46].

### Quantification of selected bacterial groups using QPCR

#### DNA extraction

Microbial genomic DNA was extracted from faecal, colonic and caecal samples using QIAamp® PowerFecal® Pro DNA Kit (Qiagen, West Sussex, UK) according to the manufacturer’s instructions. The DNA quantity and quality were evaluated using a Nanodrop spectrophotometer (Thermo Fisher Scientific, Waltham, MA, USA).

#### Bacterial primers

The domain-, function-, family- or genus-specific primers for the selected bacterial groups were available in the literature (with the exception of *Salmonella enterica*) and are provided in Table 2. The 16S rRNA gene was targeted for most bacterial groups except for *Salmonella* where the hilA gene, the transcriptional regulator of the *Salmonella* pathogenicity island 1 was selected [53] and also the butyrate-producing bacteria where the Butyryl-CoA:acetate CoA-transferase (B-CoA) gene associated with this function was selected [48, 51]. Primers were designed using two tools, Primer3 (https://primer3.org/) for larger amplicons (> 150 bp) and Primer Express™ (Applied Biosystems, Foster City, CA, USA) for smaller amplicons optimised for QPCR (< 125 bp), and their specificity was verified using Primer Basic Local Alignment Search Tool (Primer-BLAST), https://www.ncbi.nlm.nih.gov/tools/primer-blast/index.cgi.

**Table 2:** List of forward and reverse primers used for the bacterial quantification by QPCR

Target bacterial group	Forward primer 5′ to 3′ Reverse primer 5′ to 3’	Amplicon length, bp	Tm, °C	References
*Salmonella enterica*	F: TACTCAACATGGACGGCTCC R: TTTGCAAGAGAGAAGCGGGT	630	59.3 57.3	This study
Total bacteria	F: GTGCCAGCMGCCGCGGTAA R: GACTACCAGGGTATCTAAT	291	64.2 52.4	[47]
*Lactobacillus* spp.	F: AGCAGTAGGGAATCTTCCA R: CACCGCTACACATGGAG	341	54.5 55.2	[48]
*Bifidobacterium* spp.	F: GCGTGCTTAACACATGCAAGTC R: CACCCGTTTCCAGGAGCTATT	125	60.3 59.8	[49]
Enterobacteriaceae	F: ATGTTACAACCAAAGCGTACA R: TTACCYTGACGCTTAACTGC	185	54.0 56.3	[50]
Butyryl-CoA:acetate CoA-transferase (B-CoA)	F: GCIGAICATTTCACITGGAAYWSITGGCAYATG R CCTGCCTTTGCAATRTCIACRAANGC	530	67.0 64.0	[51]
*Prevotella* spp.	F: CACRGTAAACGATGGATGCC R: GGTCGGGTTGCAGACC	514	58.3 56.9	[52]

*bp*, Base pairs; *Tm*, Melting temperature

#### Plasmid preparation and QPCR for absolute quantification

The quantification of the selected bacterial groups using QPCR and the preparation of specific plasmids (total bacteria, *Lactobacillus* spp., *Bifidobacterium* spp., *Prevotella* spp., Enterobacteriaceae) to obtain the standard curves was carried out as described by Venardou et al. [39]. Additionally, plasmids containing the *hilA* and B-CoA genes were prepared from genomic DNA of *S.* Typhimurium extracted from pure cultures (DNeasy® Blood & Tissue kit, Qiagen, West Sussex, UK) and *Faecalibacterium prausnitzii* (DSMZ 17677) purchased from Leibniz Institute DSMZ-German Collection of Microorganisms and Cell Cultures (Braunschweig, Germany) respectively. The primers and genomic locations of all targeted genes that were incorporated into plasmids are outlined in Table S1 (Additional file 1). The plasmids were quantified spectrophotometrically and the copy number/μL was determined using an online tool which employs the formula mol/g × molecules/mol = molecules/g using Avogadro’s constant, 6.022 × 10^23^ molecules/mole (http://cels.uri.edu/gsc/cndna.html). The QPCR reaction (20 μL) included 3 μL template DNA, 1 μL or 2 μL (for B-CoA) of each primer (10 μmol/L), 5 μL or 3 μL (for B-CoA) nuclease-free water and 10 μL of GoTaq® qPCR Master Mix (Promega, Madison, WI, USA). All QPCR reactions were performed in duplicate on the 7500 ABI Prism Sequence Detection System (Applied Biosystems, Foster City, CA, USA) with the following cycling conditions; a denaturation step of 95 °C for 10 min, 40 cycles of 95 °C for 15 s and 60 °C for 1 min. Dissociation curves were generated to confirm the specificity of the amplicons. The efficiency of each QPCR assay was established from the slope of the curve derived from plotting the Cycle threshold (Ct) obtained from 5-fold serial dilutions of the plasmid against their arbitrary quantities. Only assays exhibiting 90–110% efficiency and generating specific products were used in this study. Bacterial counts were determined from the standard curve derived from the mean Ct value and the log transformed gene copy number of the plasmid and expressed as Log transformed gene copy number per gram of faeces or digesta (logGCN/g faeces or digesta).

### Gene expression

#### RNA extraction and cDNA synthesis

Total RNA was extracted from ileal and colonic tissue using TRI Reagent® (Sigma-Aldrich, St. Louis, MO, USA) and purified using GenElute™ Mammalian Total RNA Miniprep Kit (Sigma-Aldrich, St. Louis, MO, USA) and a DNase removal step (On-Column DNase I Digestion Set, Sigma-Aldrich, St. Louis, MO, USA) according to the manufacturers’ instructions. The quantity and purity (260/280 nm absorbance ratio ≥ 2.0) of the total RNA was determined using a Nanodrop spectrophotometer. The complimentary DNA (cDNA) was synthesised from 2 μg total RNA using the High Capacity cDNA Reverse Transcription Kit (Applied Biosystems, Foster City, CA, USA) following the manufacturer’s instructions. The total reaction volume (20 μL) was adjusted to 400 μL using nuclease-free water.

#### QPCR for relative quantification

The QPCR reaction mix (20 μL) contained 10 μL GoTaq® qPCR Master Mix, 1.2 μL forward and reverse primers (5 μmol/L), 3.8 μLnuclease-free water and 5 μL cDNA. All QPCR reactions were carried out in duplicate on the 7500 ABI Prism Sequence Detection System (Applied Biosystems, Foster City, CA, USA) with the following cycling conditions; a denaturation step of 95 °C for 10 min, 40 cycles of 95 °C for 15 s and 60 °C for 1 min. All primers were designed using the Primer Express™ Software and synthesised by MWG Biotech UK Ltd. (Milton Keynes, UK) and are presented in Table 3. The sequences of the forward and reverse primers have been described and validated previously for porcine gastrointestinal tissues [29, 60, 61] except for *IL7*, *CCL20, TP53*, *STAT3*, *CHRM1*, *NOX1* and *DUOX2* genes for which the primer pairs were newly designed, and their specificity was verified in silico using Primer-BLAST. Dissociation curves were generated to confirm the specificity of the resulting PCR products. The efficiency of each QPCR reaction was established by plotting the Ct derived from 4-fold serial dilutions of cDNA against their arbitrary quantities. Assays exhibiting 90–110% efficiency and single products were solely used in this study. Normalised relative quantities were obtained using the qbase™ PLUS software (Biogazelle, Ghent, Belgium) from two stable housekeeping reference genes, *GAPDH* and *PPIA* for the ileum and *B2M* and *PPIA* for the colon. These genes were selected as reference genes due to their lowest stability M value (< 1.5) generated by the geNorm application.

**Table 3:** Panel of target genes evaluated in the ileum and colon

Target gene	Accession No.	Forward primer 5’ to 3′ Reverse primer 5′ to 3’	Amplicon length, bp	Tm, °C
Immune response				
*IL1A*	NM_214029.1	F: CAGCCAACGGGAAGATTCTG R: ATGGCTTCCAGGTCGTCAT	76	63.0 60.5
*IL6*	NM_214399.1	F: GACAAAGCCACCACCCCTAA R: CTCGTTCTGTGACTGCAGCTTATC	69	59.8 62.7
*IL7*	NM_214135.2	F: GAGTGACTATGGGCGGTGAGA R: GCGGGCGTGGTCATGA	63	61.8 56.9
*CXCL8*	NM_213867.1	F: TGCACTTACTCTTGCCAGAACTG R: CAAACTGGCTGTTGCCTTCTT	82	61.9 61.7
*IL10*	NM_214041.1	F: GCCTTCGGCCCAGTGAA R: AGAGACCCGGTCAGCAACAA	71	63.4 63.1
*IL17A*	NM_001005729.1	F: CCCTGTCACTGCTGCTTCTG R: TCATGATTCCCGCCTTCAC	57	60.6 60.4
*IL22*	XM_001926156.1	F: GATGAGAGAGCGCTGCTACCTGG R: GAAGGACGCCACCTCCTGCATGT	112	66.0 66.0
*IFNG*	NM_213948.1	F: TCTAACCTAAGAAAGCGGAAGAGAA R: TTGCAGGCAGGATGACAATTA	81	61.1 61.5
*TNF*	NM_214022.1	F: TGGCCCCTTGAGCATCA R: CGGGCTTATCTGAGGTTTGAGA	68	62.5 62.8
*TGFB1*	NM_214015.1	F: AGGGCTACCATGCCAATTTCT R: CGGGTTGTGCTGGTTGTACA	101	60.6 61.7
*FOXP3*	NM_001128438.1	F: GTGGTGCAGTCTCTGGAACAAC R: AGGTGGGCCTGCATAGCA	68	60.6 61.2
*CCL20*^*a*^	NM_001024589.1	F: GCTCCTGGCTGCTTTGATG R: TTGCTTGCTGCTTCTGACTTG	66	58.8 57.9
*TLR4*	NM_001293317.1	F: TGCATGGAGCTGAATTTCTACAA R: GATAAATCCAGCACCTGCAGTTC	140	57·1 60·6
*TP53*^*a*^	NM_213824.3	F: CCGGGTGGAAGGGAATTT R: CCACAACGCTGTGTCGAAAA	68	56.0 57.3
*STAT3*^*a*^	NM_001044580	F: TCTTGAGAAGCCAATGGAGATTG R: TGGAGGAGGCGGGACTCT	69	58.9 60.5
Intestinal integrity				
*MUC2*	AK231524	F: CAACGGCCTCTCCTTCTCTGT R: GCCACACTGGCCCTTTGT	70	63.1 62.1
*TJP1/ZO-1*	XM_005659811.1	F: TGAGAGCCAACCATGTCTTGAA R: CTCAGACCCGGCTCTCTGTCT	76	59.9 60.0
*Cholinergic receptor*				
*CHRM1*^*a*^	NM_214034.1	F: GCCATGGCCGCCTTCT R: GGTTCTCTGTCTCCCGGTAGATG	76	56.9 64.2
*NADPH oxidases*				
*NOX1*^*a*^	XM_003484140.3	F: CTTTGAAAGGATCCTCCGATTTT R: ATGGATACATGACCACCTTGGTAA	71	57.1 59.3
*DUOX2*^*a*^	NM_213999.2	F: CTGGGCCTTGACATAGATGAGAT R: GGCAAAAAGGTGTCTGAAGAAGA	108	60.6 58.9
Reference genes				
*PPIA*	NM_214353.1	F: CGGGTCCTGGCATCTTGT R: TGGCAGTGCAAATGAAAAACT	75	62.1 60.7
*B2M*	NM_213978.1	F: CGGAAAGCCAAATTACCTGAAC R: TCTCCCCGTTTTTCAGCAAAT	83	58.2 58.4
*GAPDH*	AF017079.1	F: CAGCAATGCCTCCTGTACCA R: ACGATGCCGAAGTTGTCATG	72	62.2 62.1

*bp*, Base pairs; *Tm*, Melting temperature; *IL1A*, Interleukin 1 alpha; *IL6*, Interleukin 6; *IL7*, Interleukin 7; *CXCL8*, C-X-C motif chemokine ligand 8; *IL10*, Interleukin 10; *IL17A*, Interleukin 17 alpha; *IL22*, Interleukin 22; *IFNG*, Interferon gamma; *TNF*, Tumour necrosis factor; *TGFB1*, Transforming growth factor beta 1; *FOXP3*, Forkhead box P3; *CCL20*, C-C motif chemokine ligand 20; *TLR4*, Toll-like receptor 4; *TP53*, Tumour protein p53; *STAT3*, Signal transducer and activator of transcription 3; *MUC2*, Mucin 2; *TJP1*/*ZO-1*, Tight junction protein 1/Zona occludens 1; *CHRM1*, Cholinergic receptor muscarinic 1; *NOX1*, Nicotinamide adenine dinucleotide phosphate (NADPH) oxidase 1; *DUOX2*, Dual oxidase 2; *PPIA*, Peptidylprolyl isomerase A; *B2M*, Beta-2-microglobulin; *GAPDH*, Glyceraldehyde-3-phosphate dehydrogenase

^a^Genes encoding proteins with an established role in facilitating or inhibiting *Salmonella* infection. These proteins are associated with chemotaxis (*CCL20*), production of reactive oxygen species (*NOX1*, *DUOX2*) anti-inflammatory activity (*STAT3*, *CHRM1*) and cell survival and death (*TP53*) [54–59]

### Statistical analysis

All data was initially checked for normality using PROC UNIVARIATE procedure of Statistical Analysis Software (SAS) 9.4 (SAS Institute, Cary, NC, USA). The bacterial counts from the pure culture growth assays were analysed using PROC GLM procedure for the presence of linear and quadratic effects of ANE concentration. The biological replicate was the experimental unit. The LSMEANS statement was additionally used to calculate the least-square mean values and the standard error of the means (SEM). The performance data from the newly weaned pig experiment, FS data from both experiments and *Salmonella* shedding data from the challenge experiment were analysed by repeated measures analysis using PROC MIXED procedure of SAS [62]. The model included the fixed effects of treatment and time and their associated interaction. For the performance data, the initial weight was used as a covariate. *Salmonella* shedding data from the newly weaned pig experiment, performance data from the challenge experiment, bacterial populations data and gene expression data were analysed using PROC GLM procedure of SAS. The Bonferroni adjustment was used in the analysis of the gene expression data. The model assessed the effect of treatment with the experimental unit being the pen for the performance data and the animal within the pen for the bacterial populations and gene expression data. For the performance data, the body weight on d 25 was used as a covariate. Probability values of < 0.05 denote statistical significance. Results are presented as least-square mean values ± SEM.

## Results

### In vitro effects of ANE on *S.* Typhimurium and *B. thermophilum* growth

**Fig. 1:**
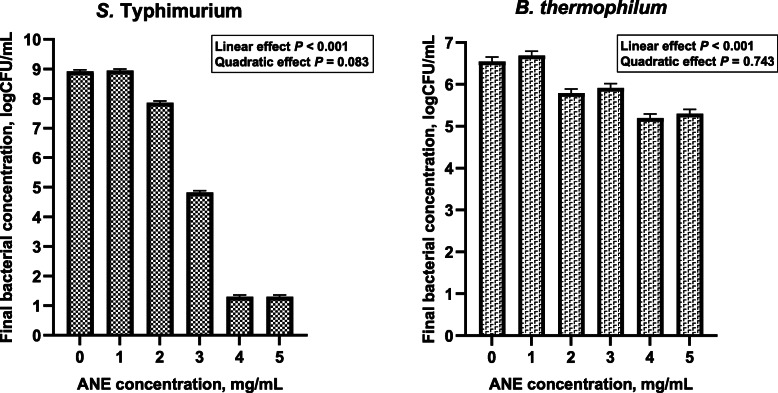
*S. * Typhimurium and *B. thermophilum* counts in response to the increasing ANE concentrations in the pure culture growth assays (Least-square mean values ± SEM). ANE, *Ascophyllum nodosum* extract; CFU, colony-forming unit

The effects of ANE on the counts of *S. *Typhimurium and *B. thermophilum* were evaluated in pure culture growth assays and presented in Fig. 1. There was a linear decrease in the counts of *S.* Typhimurium (≤ 7.6 logCFU/mL reduction) and *B. thermophilum* (≤ 1.4 logCFU/mL reduction) in response to the increasing ANE concentrations (*P* <  0.05). However, the antibacterial effect of ANE was more pronounced for *S. *Typhimurium than for *B. thermophilum* (7.6 vs. 1.4 logCFU/mL reduction, respectively).

### *Salmonella* presence and isolated serotypes in pigs on d 0

All 19 pigs sampled on d 0 were identified as *Salmonella*-positive using QPCR with average counts of 7.41 (SD 0.308) logGCN/g faeces. *Salmonella* presence was additionally confirmed using standard ISO protocols and the prevalent serotypes were identified. 11 out of the 19 pigs were *Salmonella* positive with *S.* Enteritidis being the predominant serotype (8 out of 11 pigs) followed by the monophasic variant of *S.* Typhimurium, *S.* 4,[5],12:i:- (3 out of 11 pigs).

### Newly weaned pig experiment (d 0–21)

#### Pig performance and faecal consistency

The effects of ZnO and the two ANE concentrations on final body weight (BW), average daily gain (ADG), average daily feed intake (ADFI) and gain to feed ratio (G:F) are presented in Table 4. There was no time × treatment interaction on final BW, ADG, ADFI and G:F (*P* > 0.05). However, during the overall 21-day experimental period, dietary supplementation with ZnO increased final BW, ADG and ADFI compared to all other treatments (*P* <  0.05), whereas none of the ANE concentrations had an effect on these parameters (*P* > 0.05). Both ZnO and high ANE supplementation increased G:F compared to the other treatments (*P* <  0.05).

**Table 4:** Effects of dietary treatments on pig performance in the newly weaned pig experiment (Least-square mean values ± SEM)

	Treatment^**1**^				SEM	Time, d			SEM	Treatment effect	Time effect	Treatment × Time effect
	Control	ZnO	Low ANE	High ANE		7	14	21				
**Final BW, kg**	15.0^b^	16.2^a^	14.5^b^	15.2^b^	0.31	9.0^C^	11.3^B^	15.2^A^	0.16	< 0.001	< 0.001	0.432
**ADG, kg/d**	0.29^b^	0.37^a^	0.29^b^	0.32^b^	0.016	0.09^C^	0.32^B^	0.54^A^	0.014	0.001	< 0.001	0.644
**ADFI, kg/d**	0.50^b^	0.56^a^	0.50^b^	0.51^b^	0.015	0.20^C^	0.55^B^	0.80^A^	0.013	0.003	< 0.001	0.166
**G:F ratio**	0.51^b^	0.62^a^	0.51^b^	0.61^a^	0.034	0.43^C^	0.59^B^	0.68^A^	0.029	0.017	< 0.001	0.186

*ANE*, *Ascophyllum nodosum* extract; *ZnO*, Zinc oxide; *BW*, Body weight; *ADG*, Average daily gain; *ADFI*, Average daily feed intake; *G:F*; Gain to feed ratio

^A,B,C^Mean values within a row with different superscript capital letter indicate significant differences between days (*P* < 0.05)

^1^A total of 12 replicates were used per dietary treatment (replicate = pen)

**Fig. 2:**
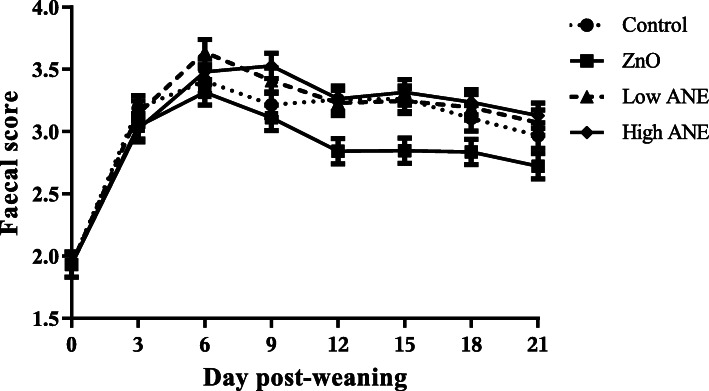
Effect of dietary treatments on faecal consistency during the first 21 d post-weaning. The scoring system was from 1 to 5: (1) hard, firm faeces; (2) slightly soft faeces; (3) soft, partially formed faeces; (4) loose, semi-liquid faeces; (5) watery, mucous like faeces [43]. Data are expressed as least-square mean values ± SEM represented in vertical bars. A total of 12 replicates were used per dietary treatment (replicate = pen). ANE, *Ascophyllum nodosum* extract; ZnO, Zinc oxide

The effects of ZnO and the two ANE concentrations on FS are presented in Fig. 2. There was no time × treatment interaction on FS (*P* > 0.05). Overall, dietary supplementation with ZnO reduced FS compared to all other treatments during the 21-day experimental period [2.83 (ZnO) vs. 3.04 (control), 3.11 (low ANE) and 3.11 (high ANE) ± 0.036, *P* <  0.05]. None of the ANE concentrations had an effect on FS (*P* > 0.05).

#### *Salmonella* faecal shedding

The effects of ZnO and ANE concentrations on *Salmonella* faecal shedding are presented in Table 5. On d 14, there was no effect of the dietary treatments on *Salmonella* counts in the faeces (*P* > 0.05). However, on d 21, dietary supplementation with high ANE reduced *Salmonella* counts in the faeces compared to the control and low ANE group (*P* <  0.05).

**Table 5:** Effects of dietary treatments on *Salmonella* shedding in naturally infected weaned pigs (Least-square mean values ± SEM)

	Day	Treatment^**1**^				SEM	***P***-value
		Control	ZnO	Low ANE	High ANE		
**Faecal** ***Salmonella*** **counts, logGCN/g faeces**	14	6.74	6.78	6.97	6.84	0.115	0.475
	21	7.25^a^	7.02^ab^	7.29^a^	6.70^b^	0.121	0.006

*ANE*, *Ascophyllum nodosum* extract; *ZnO*, Zinc oxide; *GCN*, Gene copy number

^a,b^Mean values within a row with different superscript lowercase letter indicate significant differences between dietary treatments (*P* < 0.05)

^1^A total of 12 replicates were used per dietary treatment (replicate = pig)

### Challenge experiment (d 25–34)

#### Pig performance and faecal consistency

The effect of ANE and the residual effect of ZnO on final BW, ADG, ADFI and G:F are presented in Table 6. There was no effect of the dietary treatments on final BW, ADG, ADFI and G:F during the 9-day period (*P* > 0.05). There were no treatment and time effects and time × treatment interaction on FS (*P* > 0.05, data not shown).

**Table 6:** Effect of ANE and residual effect of ZnO on pig performance in the challenge experiment (Least-square mean values ± SEM)

	Treatment^**1**^			SEM	***P***-value
	Control	ZnO-residual	ANE		
**Final BW, kg**	25.1	24.6	24.7	0.34	0.520
**ADG, kg/d**	0.75	0.69	0.71	0.038	0.517
**ADFI, kg/d**	1.13	1.15	1.10	0.027	0.418
**G:F**	0.67	0.61	0.64	0.024	0.306

*ANE*, *Ascophyllum nodosum* extract; *ZnO*, Zinc oxide; *BW*, Body weight; *ADG*, Average daily gain; *ADFI*, Average daily feed intake; *G:F*, Gain to feed ratio

^1^A total of 6 replicates were used per dietary treatment (replicate = pen)

#### *Salmonella* faecal shedding

The effect of ANE and the residual effect of ZnO on *Salmonella* faecal shedding following the experimental re-infection with *S.* Typhimurium are presented in Table 7. The dietary treatments had no effect on *Salmonella* counts in the faeces on any of the days tested (*P* > 0.05). There was a time effect on *Salmonella* shedding. *Salmonella* counts were lower in the faeces on d 25 compared to d 34 (7.13 logGCN/g faeces vs. 7.35 logGCN/g faeces ± 0.150, *P* <  0.05).

**Table 7:** Effect of ANE and residual effect of ZnO on *Salmonella* shedding following experimental re-infection with *S.* Typhimurium (Least-square mean values ± SEM)

Time, d	Treatment^**1**^	Faecal *Salmonella* counts, logGCN/g faeces
25	Control	7.22
	ZnO-residual	7.16
	ANE	7.02
27	Control	7.33
	ZnO-residual	7.24
	ANE	7.24
34	Control	7.41
	ZnO-residual	7.34
	ANE	7.30
**SEM**		0.171
***P*-value**		
Treatment effect		0.262
Time effect		0.025
Treatment × Time effect		0.961

*ANE*, *Ascophyllum nodosum* extract; *ZnO*, Zinc oxide; *GCN*, Gene copy number

^1^A total of 12 replicates were used per dietary treatment (replicate = pig)

#### Enumeration of *Salmonella* in colonic and caecal digesta and selected bacterial populations in the colonic digesta

The effect of ANE and the residual effect of ZnO on *Salmonella* counts in colonic and caecal digesta and on selected colonic bacterial populations of pigs on d 34 are presented in Table 8. There was no effect of the dietary treatments on *Salmonella* counts in colonic and caecal digesta (*P* > 0.05). Enterobacteriaceae counts were decreased in the ANE-supplemented and ZnO-residual groups compared to the control group (*P* < 0.05). Dietary supplementation with ANE increased *Bifidobacterium* spp. counts compared to the ZnO-residual group (*P* < 0.05), but not compared to the control group (*P* = 0.112). There was no effect of the dietary treatments on the counts of total bacteria, *Lactobacillus* spp., butyrate-producing bacteria (B-CoA) and *Prevotella* spp. (*P* > 0.05).

**Table 8:** Effect of ANE and residual effect of ZnO on colonic and caecal *Salmonella* counts and on colonic bacterial populations on d 34 (Least-square mean values ± SEM)

Bacterial group, logGCN/g digesta	Treatment^**1**^			SEM	***P***-value
	Control	ZnO-residual	ANE		
*Caecal digesta*					
*Salmonella enterica*	6.16	6.30	6.28	0.093	0.536
*Colonic digesta*					
Total bacteria	11.98	11.95	11.87	0.051	0.324
*Lactobacillus* spp.	10.97	11.07	11.28	0.146	0.324
*Bifidobacterium* spp.	7.12^ab^	6.85^b^	7.49^a^	0.159	0.025
Enterobacteriaceae	7.35^a^	6.49^b^	6.66^b^	0.210	0.017
B-CoA	8.08	8.18	8.12	0.112	0.809
*Prevotella* spp.	11.62	11.57	11.58	0.075	0.884
*Salmonella enterica*	7.15	7.17	6.98	0.098	0.321

*ANE*, *Ascophyllum nodosum* extract; *ZnO*, Zinc oxide; *GCN*, Gene copy number; *B-CoA*, Butyryl-CoA:acetate CoA-transferase

^a,b^Mean values within a row with different lowercase superscript letter indicate significant differences between dietary treatments (*P* < 0.05)

^1^A total of 12 replicates were used per dietary treatment (replicate = pig)

#### Gene expression in ileum and colon

The effect of ANE and the residual effect of ZnO on the expression of selected genes in the ileum and colon of pigs are presented in Table 9. In the ileum, dietary supplementation with ANE decreased the expression of Interleukin 22 (*IL22*) and Transforming growth factor beta 1 (*TGFB1*) compared to the control (*P* < 0.05). In the colon, dietary supplementation with ANE decreased the expression of C-C motif chemokine ligand 20 (*CCL20*) compared to the ZnO-residual group (*P* < 0.05).

**Table 9:** Effect of ANE and residual effect of ZnO on the expression of inflammation-associated genes in the ileum and colon (Least-square mean values ± SEM)

Target gene	Treatment			SEM	***P***-value
	Control	ZnO-residual	ANE		
**Ileum**^**1**^					
*IL1A*	1.01	0.90	0.96	0.056	0.384
*IL6*	1.18	1.01	1.01	0.141	0.625
*IL7*	1.23	1.01	1.01	0.133	0.330
*CXCL8*	1.47	1.09	0.95	0.320	0.555
*IL10*	0.98	0.81	0.99	0.058	0.075
*IL17A*	1.28	1.68	0.75	0.343	0.116
*IL22*	1.47^a^	0.86^ab^	0.72^b^	0.190	0.017
*IFNG*	1.31	1.17	0.99	0.245	0.691
*TNF*	1.00	1.17	0.99	0.116	0.474
*TGFB1*	1.25^a^	0.99^ab^	0.86^b^	0.083	0.007
*FOXP3*	1.05	0.97	0.97	0.125	0.882
*CCL20*	1.24	1.13	0.80	0.209	0.327
*TLR4*	1.10	0.96	1.02	0.096	0.571
*TP53*	0.98	1.08	1.03	0.052	0.356
*MUC2*	1.05	0.94	1.23	0.195	0.346
*TJP1/ZO-1*	1.05	1.06	1.06	0.094	0.905
*STAT3*	1.39	1.07	1.09	0.206	0.979
*CHRM1*	1.29	1.35	1.54	0.459	0.717
*NOX1*	1.27	1.21	0.96	0.266	0.368
*DUOX2*	0.88	0.80	0.75	0.096	0.636
**Colon**^**2**^					
*IL1A*	1.17	0.95	0.96	0.122	0.420
*IL6*	1.14	1.05	1.25	0.221	0.825
*IL7*	1.07	0.94	1.08	0.113	0.514
*CXCL8*	1.12	1.13	0.94	0.138	0.574
*IL10*	1.09	1.00	1.03	0.125	0.882
*IL17A*	1.10	1.03	1.02	0.172	0.940
*IL22*	1.24	0.91	1.23	0.213	0.475
*IFNG*	0.91	1.00	1.04	0.097	0.642
*TNF*	1.20	0.97	0.90	0.122	0.196
*TGFB1*	1.07	0.89	1.16	0.117	0.289
*FOXP3*	0.88	1.01	1.28	0.153	0.196
*CCL20*	1.13^ab^	1.67^a^	0.62^b^	0.223	0.007
*TLR4*	0.91	0.98	1.13	0.114	0.154
*TP53*	1.03	0.99	0.98	0.055	0.752
*MUC2*	1.09	1.06	1.11	0.105	0.953
*TJP1/ZO-1*	0.99	1.05	1.00	0.044	0.529
*STAT3*	1.10	0.98	1.17	0.120	0.521
*CHRM1*	1.20	1.50	1.24	0.235	0.623
*NOX1*	1.09	1.13	1.08	0.114	0.959
*DUOX2*	1.05	1.23	1.07	0.146	0.630

*ANE*, *Ascophyllum nodosum* extract; *ZnO*, Zinc oxide; *IL1A*, Interleukin 1 alpha; *IL6*, Interleukin 6; *IL7*, Interleukin 7; *CXCL8*, C-X-C motif chemokine ligand 8; *IL10*, Interleukin 10; *IL17A*, Interleukin 17 alpha; *IL22*, Interleukin 22; *IFNG*, Interferon gamma; *TNF*, Tumour necrosis factor; *TGFB1*, Transforming growth factor beta 1; *FOXP3*, Forkhead box P3; *CCL20*, C-C motif chemokine ligand 20; *TLR4*, Toll-like receptor 4; *TP53*, Tumour protein p53; *MUC2*, Mucin 2; *TJP1*/*ZO-1*, Tight junction protein 1/Zona occludens 1; *STAT3*, Signal transducer and activator of transcription 3; *CHRM1*, Cholinergic receptor muscarinic 1; *NOX1*, Nicotinamide adenine dinucleotide phosphate (NADPH) oxidase 1; *DUOX2*, Dual oxidase 2

^a,b^Mean values within a row with different superscript lowercase letter indicate significant differences between dietary treatments (*P* < 0.05)v

^1^A total of 12 replicates were used per dietary treatment apart from ANE whereby 11 replicates were used (replicate = pig)

^2^A total of 12 replicates were used per dietary treatment (replicate = pig)

## Discussion

In this study, the fucoidan-rich *A. nodosum* extract (ANE) had a strong concentration-dependent anti-*S.* Typhimurium activity in vitro and was therefore further explored in two in vivo experiments. In a ‘newly weaned pig’ experimental model with naturally infected pigs, high ANE supplementation reduced *Salmonella* counts in the faeces on d 21 post-weaning and increased G:F. In a ‘challenge’ experiment, none of the dietary treatments had an effect on performance or *Salmonella* counts in the faeces or digesta. Nevertheless, ANE supplementation reduced the Enterobacteriaceae counts in the colon compared to the control group, with no effect on the other bacterial populations measured. Additionally, the expression of *IL22* and *TGFB1* was decreased in the ileum of the ANE-supplemented pigs compared to the control group. These results indicate that ANE may be a promising dietary supplement with which to counteract the negative impact of *Salmonella* infection on the gastrointestinal microbiota and inflammation in pigs and could be usefully combined with other husbandry measures. An additional finding of this study was the reduced Enterobacteriaceae counts in the ZnO-residual group indicating that short-term ZnO supplementation could have longer lasting residual effects on the gastrointestinal microbiota.

In the pure culture growth assay, the inclusion of ANE led to a significant and concentration-dependent reduction in the counts of the pathogen *S. *Typhimurium. ANE also reduced the counts of the commensal *B. thermophilum*, though to a lesser extent. While it is not possible to definitively identify which is the bioactive component of the ANE extract, it consisted predominantly of fucoidan (~ 47%), with other polysaccharides including laminarin, mannitol and alginate present at lower concentrations (18.6%, 10.7% and 4.6%, respectively). Depolymerised fucoidans are thought to exert antibacterial activity by disrupting the integrity and permeability of the bacterial cell membrane resulting in cell leakage and death and/or by nutrient trapping [18, 19, 63]. We hypothesise that a mixture of low molecular weight fucoidans with anti-*Salmonella* activity are present in the ANE as a result of fucoidan depolymerisation due to the HCl and high temperature of the hydrothermal-assisted extraction methodology [32, 64].

The immediate post-weaning period in commercial pig production systems is characterised by reduced growth, reduced feed intake and an increased incidence of diarrhoea [1, 2]. *Salmonella* infection and shedding in pigs has been associated with similar suboptimal measures of performance [65–68]. In the newly weaned pig experiment, dietary supplementation with high ANE improved G:F and reduced faecal *Salmonella* counts. While the ANE displayed a strong anti-*S.* Typhimurium activity during its in vitro evaluation in the current study, the reduction in *Salmonella* counts in vivo was not of the same magnitude. This may be due in part to the fact that the depolymerised fractions of fucoidan, previously hypothesised to exert the antibacterial activity, may have increased availability as a substrate for fermentation to different members of the gastrointestinal microbiota as observed elsewhere [20, 69] which might explain the reduced anti-*S.* Typhimurium activity of ANE in vivo.

The influence of ANE supplementation was further explored in pigs during the grower phase. At the start of this phase, the pigs were moved and regrouped and received an experimental *Salmonella* infection. Even though a slight increase in *Salmonella* shedding was observed, it appears that the re-infection with *S.* Typhimurium did not have a major impact on either the established *Salmonella* population or *Salmonella* shedding. Despite the reduced *Salmonella* counts in the faeces of ANE-supplemented pigs in the newly weaned pig experiment, this observation was not evident in the challenge experiment, either in the faeces collected at different time points or in the colonic and caecal digesta collected at the end of the experiment. In a previous study, dietary supplementation with a fucoidan-rich seaweed extract reduced the faecal, colonic and caecal *Salmonella* counts in grower pigs experimentally infected with *S.* Typhimurium [29]. However, in that study of Bouwhuis et al. [29], the supplementation of the seaweed extract preceded the *S.* Typhimurium infection in pigs, whereas in the current study the pigs had a natural *Salmonella* infection with two different serotypes (*S.*Enteritidis  and *S.* 4,[5],12:i:-) prior to the ANE supplementation. These results suggest that early ANE supplementation might have been more effective as a preventative dietary intervention for *Salmonella* infection in pigs. In addition, there may be variation in the antibacterial activity of ANE against different *Salmonella* serotypes. Nevertheless, ANE supplementation was associated with reduced Enterobacteriaceae counts in the colonic digesta with no negative impact on the counts of the other bacterial populations measured, namely total bacteria, *Lactobacillus* spp., *Bifidobacterium* spp., *Prevotella* spp. and butyrate-producing bacteria. This finding suggests that ANE has antibacterial activity against various members within the Enterobacteriaceae family, thus, limiting the available fucoidan that could target the *Salmonella* subpopulation. The lower Enterobacteriaceae counts could be indicative of a healthier composition of the colonic microbiota in the ANE-supplemented pigs, as this bacterial family is considered a marker of dysbiosis and a predisposing factor for intestinal disease post-weaning [3, 70, 71].

*Salmonella* infection in pigs is accompanied by intestinal inflammation that has a negative impact on the composition of the residing microbiota, thus, facilitating pathogen colonisation and shedding [68, 72]. Dietary supplementation with ANE altered the mucosal immune response in the ileum by reducing the expression of *IL22* and *TGFB1*. Several murine studies have demonstrated that elevated *IL22* expression and protein synthesis are associated with increased susceptibility to *Salmonella* colonisation and persistent infection in the gastrointestinal tract due to the IL22-induced suppression of commensal bacteria via the secretion of antimicrobial proteins (lipocalin-1, S100A8, S100A9, Reg3β, Reg3γ) [73–75]. IL22 may also contribute to *Salmonella* colonisation and the carrier state in pigs as its production was increased following experimental *Salmonella* infection [76]. The reduction in *IL22* expression may be linked to the reduced *TGFB1* expression, as TGFβ1 promotes the differentiation of Th17 cells that produce IL22 [77, 78]. Previous studies have characterised the inhibitory effect of fucoidan on TGFβ1 production and activity by interfering with TGFβ1 activation and binding to its receptor [79, 80]. Furthermore, *TGFB1* gene expression was elevated in mice with chronic *S.* Typhimurium colonisation [81], whereas reduced TGFβ1 presence decreased *S.* Typhimurium counts in both the spleen and liver, highlighting its potential role in pathogen persistence [82]. Thus, ANE supplementation may have the potential to decrease the immune responses that facilitate *Salmonella* colonisation and persistence, as evidenced by the reduced *IL22* and *TGFB1* expression.

Dietary supplementation with the pharmacological dose of ZnO during the first 21 d post-weaning, improved growth performance and faecal consistency similar to previous weaned pig studies with [83, 84] or presumably without [12, 85, 86] a concurrent *Salmonella* infection. In the challenge experiment, no differences in the counts of most bacterial populations were observed between the control and ZnO-residual groups two weeks post ZnO removal, which is in agreement with the assumption of Janczyk et al. [87] that the impact of ZnO on the composition and activity of the gastrointestinal microbiota was reversible after ZnO withdrawal. However, Enterobacteriaceae counts were significantly lower compared to the control indicating that ZnO may have a long-term residual impact on the different members of the microbiota. The observed reduction may be attributed to the release of Zn^2^^+^ ions from the liver and kidneys, as Zn concentration was reported to still be high, albeit decreasing, in these tissues two weeks after ZnO removal [87]. However, prolonged exposure to high Zn levels could potentially increase Zn resistance [88] and AMR [14, 16] within the *Escherichia coli* population as indicated in previous studies. The residual effect of ZnO on the Enterobacteriaceae counts observed in the current study merits further research concerning the prevalence of Zn resistance and AMR within this family following ZnO removal, particularly in countries where ZnO supplementation is still applicable during the weaning transition.

We hypothesise that the observed beneficial effects on *Salmonella* shedding, the composition of the colonic microbiota and the expression of inflammatory genes in the ileum of the ANE-supplemented pigs were associated with a mixture of low molecular weight fucoidans, as it was the principal component (~ 47%) in this extract. Nevertheless, this should be confirmed by future experiments with purified fucoidan fractions of ANE. Furthermore, the potential contributing role of other components of ANE such as laminarin (~ 19% in ANE) on the observed bioactivities should also be explored. In previous studies, laminarin supplementation led to reduced Enterobacteriaceae counts and expression of inflammatory markers in the gastrointestinal tract of weaned pigs [89–91]. This polysaccharide was also present in moderate amount in the *A. nodosum* extract supplemented to the pigs in the Salmonella study of Bouwhuis et al. [29].

## Conclusion

In conclusion, the anti-*Salmonella* activity of ANE was established in vitro prior to its in vivo evaluation in two consecutive experiments in naturally infected weaned pigs. In the newly weaned pig experiment, high ANE supplementation improved G:F post-weaning while also reducing faecal *Salmonella* counts. In the challenge experiment, a slight increase in *Salmonella* shedding was observed in response to pig transfer to the grower houses, regrouping and experimental re-infection with *S. *Typhimurium.  ANE supplementation had no effect on *Salmonella* counts; nevertheless, it reduced Enterobacteriaceae counts, as well as the expression of the inflammatory *IL22* and *TGFB1* which are associated with colonisation and persistent *Salmonella* infection. Thus, the use of ANE as a dietary supplement merits further exploration regarding its potential to prevent *Salmonella* colonisation and persistence in *Salmonella*-free pigs and to alleviate the gastrointestinal dysfunction in newly weaned pigs. In this study, a potential long-term residual effect of ZnO on the gastrointestinal tract was indicated by the reduced Enterobacteriaceae counts that should be further investigated regarding its implications on pig health.

## Supplementary Information

**Additional file 1: Table S1.** List of 16S rRNA regions incorporated into plasmids for the preparation of specific *Escherichia coli* clones used for the quantification of *Salmonella enterica*, *Lactobacillus* spp., total bacteria, Enterobacteriaceae, *Bifidobacterium* spp., *Prevotella* spp. and butyrate-producing bacteria.

## Data Availability

All data generated and/or analysed during this study are available from the corresponding author upon reasonable request.

## Abbreviations

ADFI: Average daily feed intake
ADG: Average daily gain
AMR: Antimicrobial resistance
ANE: *Ascophyllum nodosum* extract
B2M: Beta-2-microglobulin
B-CoA: Butyryl-CoA:acetate CoA-transferase
bp: Base pairs
BW: Body weight
CCL20: C-C motif chemokine ligand 20
cDNA: Complimentary DNA
CFU: Colony-forming unity
CHRM1: Cholinergic receptor muscarinic 1
Ct: Cycle threshold
CXCL8: C-X-C motif chemokine ligand 8
DUOX2: Dual oxidase 2
FOXP3: Forkhead box P3
FS: Faecal scores
GAPDH: Glyceraldehyde-3-phosphate dehydrogenase
GCN: Gene copy number
G:F: Gain to feed ratio
IFNG: Interferon gamma
IL: Interleukin
ISO: International Organisation for Standardization
MRS: de Man, Rogosa and Sharpe broth
MUC2: Mucin 2
NOX1: Nicotinamide adenine dinucleotide phosphate (NADPH) oxidase 1
PPIA: Peptidylprolyl isomerase A
QPCR: Quantitative real time polymerase chain reaction
SAS: Statistical Analysis Software
SD: Standard deviation
SEM: Standard error of the means
STAT3: Signal transducer and activator of transcription 3
TGFB1: Transforming growth factor beta 1
TJP1/ZO-1: Tight junction protein 1/Zona occludens 1
TLR4: Toll-like receptor 4
Tm: Melting temperature
TNF: Tumour necrosis factor
TP53: Tumour protein p53
TSB: Tryptone soya broth
ZnO: Zinc oxide
