# Supplementary material for: Potential of a fucoidan-rich Ascophyllum nodosum extract to reduce Salmonella shedding and improve gastrointestinal health in weaned pigs naturally infected with Salmonella
Source: J Anim Sci Biotechnol. 2022 Apr 4;13:39. doi: 10.1186/s40104-022-00685-4 (PMC8978420; doi:10.1186/s40104-022-00685-4)
Supplement: Supplementary file 1 — Additional file 1: Table S1. List of 16S rRNA regions incorporated into plasmids for the preparation of specific Escherichia coli clones used for the quantification of Salmonella enterica, Lactobacillus spp., total bacteria, Enterobacteriaceae, Bifidobacterium spp., Prevotella spp. and butyrate-producing bacteria. [file 40104_2022_685_MOESM1_ESM.docx]

**Table S1** List of 16S rRNA regions incorporated into plasmids for the preparation of specific Escherichia coli clones used for the quantification of Salmonella enterica, Lactobacillus spp., total bacteria, Enterobacteriaceae, Bifidobacterium spp., Prevotella spp. and butyrate-producing bacteria

| Bacterial group | GenBank I.D. (for purposes of alignment) | Region aligned | Description | Forward primer 5' - 3'  Reverse primer 5' - 3' | Tm, °C | Amplicon length, bp |
| --- | --- | --- | --- | --- | --- | --- |
| *Salmonella enterica* | CP033360.1 | 3616758 - 3617387 | Cloned Fragment and QPCR amplicon | F: TACTCAACATGGACGGCTCC  R: TTTGCAAGAGAGAAGCGGGT | 59.3  57.3 | 630 |
| *Lactobacillus* spp. | CP017124.1 | 367650 -367990 | Cloned Fragment and QPCR amplicon | F: AGCAGTAGGGAATCTTCCA  R: CACCGCTACACATGGAG | 54.5  55.2 | 341 |
| Total bacteria | CP033387.1 | 1195141 -1195413 | Cloned Fragment and QPCR amplicon | F: GTGCCAGCMGCCGCGGTAA  R: GACTACCAGGGTATCTAAT | 64.2  52.4 | 291 |
| Enterobacteriaceae | CP041955.1 | 483389 -483554 | Cloned Fragment and QPCR amplicon | F: ATGTTACAACCAAAGCGTACA  R: TTACCYTGACGCTTAACTGC | 54.0  56.3 | 185 |
| *Bifidobacterium* spp. | CP016019.1 | 2328042 -2328627 | Cloned Fragment | F: GCAATATTCCCCACTGCTGC  R: GGTGTGGTGGTGGTTTGAGA | 59.3  59.3 | 586 |
|  |  | 2328368 -2328244 | QPCR amplicon located within the cloned fragment | F: GCGTGCTTAACACATGCAAGTC  R: CACCCGTTTCCAGGAGCTATT | 60.3  59.8 | 125 |
| *Prevotella* spp. | CP024730.1 | 246704 -246191 | Cloned Fragment and QPCR amplicon | F: CACRGTAAACGATGGATGCC  R: GGTCGGGTTGCAGACC | 58.3  56.9 | 514 |
| Butyrate-producing bacteria | Louis and Flint, 2007, Metzler-Zebeli *et al*, 2010 | - | Cloned Fragment and QPCR amplicon | F: GCIGAICATTTCACITGGAAYWSITGGCAYATG  R: CCTGCCTTTGCAATRTCIACRAANGC | 67.0  64.0 | 530 |

bp, Base pairs; Tm, Melting temperature
